# Changes in physical activity and risk of all-cause mortality in patients on maintence hemodialysis: a retrospective cohort study

**DOI:** 10.1186/s12882-017-0569-7

**Published:** 2017-05-08

**Authors:** Takahiro Shimoda, Ryota Matsuzawa, Kei Yoneki, Manae Harada, Takaaki Watanabe, Mika Matsumoto, Atsushi Yoshida, Yasuo Takeuchi, Atsuhiko Matsunaga

**Affiliations:** 1 Department of Rehabilitation Sciences, Kitasato University Graduate School of Medical Sciences, Sagamihara, Japan; 2 Department of Rehabilitation, Kitasato University Hospital, 1-15-1 Kitasato, Sagamihara, Kanagawa 252-0375 Japan; 3 Department of Hemodialysis Center, Sagami Circulatory Organ Clinic, Sagamihara, Japan; 4 Department of Nephrology in Internal Medicine, Kitasato University School of Medicine, Sagamihara, Japan

**Keywords:** Chronic renal failure, Dialysis, Exercise, Survival analysis

## Abstract

**Background:**

A previous cohort study indicated a significant association of lower baseline level of physical activity in hemodialysis patients with elevated risks of mortality. However, there have been no reports regarding the association between changes in physical activity over time and mortality in hemodialysis patients. This study was performed to examine the prognostic significance of physical activity changes in hemodialysis patients.

**Methods:**

This retrospective cohort study was performed in 192 hemodialysis patients with a 7-year follow-up. The average number of steps taken per non-dialysis day was used as a measure of physical activity. Forty (20.8%) patients had died during the follow-up period. The percentage change in physical activity between baseline and 12 months was determined, and patients were divided into three categories according to changes in physical activity. A decrease or increase in physical activity > 30% was defined as *becoming less* or *more active*, respectively, while decrease or increase in physical activity < 30% were classified as *stable*.

**Results:**

Forty seven (24.5%), 51 (26.6%), and 94 (49.0%) patients were classified as *becoming less active*, *becoming more active*, and *stable*, respectively. The hazard ratio on multivariate analysis in patients with decreased physical activity was 3.68 (95% confidence interval, 1.55–8.78; *P* < 0.01) compared to those with increased physical activity.

**Conclusions:**

Reductions in physical activity were significantly associated with poor prognosis independent of not only patient characteristics but also baseline physical activity. Therefore, improved prognosis in hemodialysis patients requires means of preventing a decline in physical activity over time.

## Background

Despite continuous progress in dialysis technology and disease management, mortality rate remains high in patients on hemodialysis. Physically inactive could contribute to mortality excess of hemodialysis patient, as hemodialysis patients are inactive compared to individuals with normal kidney function [1–4] and approximately 60% of hemodialysis patients were reported to exercise less than once a week [5]. However, routine care for hemodialysis patients does not include interventions to address this sedentary behavior.

A previous systematic review indicated a significant association between lower baseline level of physical activity and elevated risks of chronic diseases, such as cancer, diabetes, and vascular disease [6]. In addition to baseline physical activity, an inverse association was observed between decrease in physical activity over time and the risks of adverse events in subjects with impaired glucose tolerance and chronic heart failure [7, 8]. Hemodialysis patients showed an association between lower initial levels of physical activity and elevated mortality risk [9–13]. As the levels of physical activity in hemodialysis patients are affected by not only time constraints during dialysis treatment but also by aging, physical function decline, exacerbation of dialysis-related symptoms or comorbidities, depression, and other social factors, these patients would show further reductions in physical activity over time. Therefore, the effects of yearly changes in physical activity on prognosis in hemodialysis patients should be evaluated.

To our knowledge, there have been no previously reports regarding the influence of yearly changes in physical activity on prognosis in hemodialysis patients, and it is unclear whether changes in physical activity over time are associated with mortality risk. The present study was performed to examine whether changes in physical activity affected all-cause mortality in hemodialysis patients.

## Methods

### Study population

A total of 550 patients from the Hemodialysis Center, Sagami Circulatory Organ Clinic, were retrospectively enrolled in this study between October 2002 and March 2014. All patients were undergoing maintenance hemodialysis therapy three times a week, which is the most common schedule in Japan according to the Japanese Society for Dialysis Therapy. The exclusion criteria were as follows: hospitalization within 3 months prior to the study and at 12 months; recent myocardial infarction or angina pectoris; uncontrolled cardiac arrhythmia, hemodynamic instability, uncontrolled hypertension, or renal osteodystrophy with severe arthralgia; or the requirement for assistance in walking from another person. The study was approved by the Kitasato University Allied Health Sciences Research Ethics Committee.

### Demographic and clinical factors

Data on demographic factors (age, sex, and time on hemodialysis), physical constitution (body mass index), primary causes of end-stage renal disease, and comorbidities (atherosclerotic heart disease, congestive heart failure, cerebrovascular accident/transient ischemic attack (TIA), peripheral vascular disease, dysrhythmia, and other cardiac diseases, chronic obstructive pulmonary disease, gastrointestinal bleeding, liver disease, cancer, and diabetes) were collected at the time of entry into the study. Serum albumin levels were obtained from hospital charts. Comorbid illnesses were quantified using a comorbidity index developed for dialysis patients consisting of primary cause of end-stage renal disease and 11 comorbidities, and was calculated using the method described previously to analyze survival of hemodialysis patients [14].

### Physical activity

An accelerometer (Lifecorder; Suzuken Co. Ltd., Nagoya, Japan) that can continuously measure the intensity, duration, and frequency of activities was used for objective assessment of physical activity. The accuracy and reliability of this instrument were reported previously [15, 16]. In this study, the accelerometer was worn around the waist, and translated body acceleration as motion recorded as number of steps taken. The patients were instructed to wear the device continuously during waking hours for 7 days and to avoid allowing it to come into contact with water. The patients were also asked to maintain their typical weekly schedules. To ensure that measurement periods were typical of their weekly activity patterns, data were excluded when patients traveled or had an acute illness. Prior to analysis, the accelerometer data were inspected to ensure that there were no obvious errors, such as failure to acquire data or wear the device. Measurements from a period of 4 consecutive non-dialysis days were analyzed. Physical activity was reassessed using the same criteria 1 year later. The effects of changes in physical activity on survival were assessed using Cox proportional hazard models.

### Statistical analysis

The average number of steps taken per non-dialysis day was used as a measure of physical activity. The difference in physical activity between baseline and 12 months was calculated, and the percentage change in physical activity was obtained by dividing the difference between the two time points by the baseline physical activity using the following formula: [(physical activity at 12 months - physical activity at baseline)/physical activity at baseline] × 100.

A decrease or increase in physical activity > 30% was defined as becoming less or more active, respectively [7, 17], while subjects with an decrease or increase in physical activity < 30% were classified as *stable*. Hemodialysis patients were divided into three categories according to change in physical activity: (a) *becoming less active* group, (b) *stable* group, and (c) *becoming more active* group.

Demographic and clinical factors are reported by category of change in physical activity, and are presented as means ± standard deviation (SD) or number (percentage) and were tested for significance by analysis of variance (ANOVA). The χ^2^ test for trends was used to test for dose–response relations of variables between the three groups. For Kaplan–Meier estimates of survival curves, the data for the 7-year follow-up period were truncated to avoid an insufficient number of patients at risk, and the differences between the three groups were examined using the log-rank test. The independent prognostic effect of change in physical activity on survival was estimated by Cox proportional hazard regression analysis after adjustment for confounders, including age, sex, time on hemodialysis, body mass index, primary kidney disease, comorbidities, serum albumin, and baseline physical activity. We used the restricted cubic spline procedure to model the non-linear relation between change in physical activity as a continuous variable and mortality. We considered five knots for the model at the 5, 27.5, 50, 72.5, and 95th centiles. In addition, we investigated the prognostic significance of baseline physical activity. Patients were categorized into two physical activity groups by a physical activity cutoff value of 5000 steps per day [18], and the difference between groups was tested using a log-rank test. Statistical analyses were performed using R version 3.3.0 (R Foundation for Statistical Computing, Vienna, Austria) [19]. In all analyses, *P* < 0.05 was taken to indicate statistical significance.

## Results

### Baseline characteristics and changes in physical activity

**Fig. 1:**
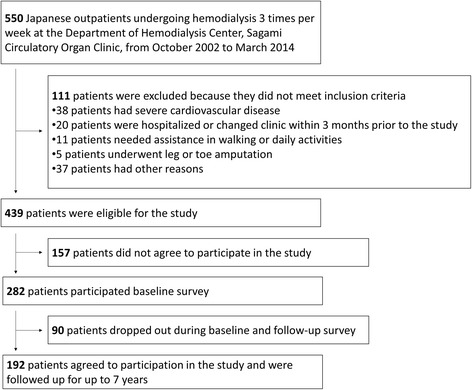
Flow diagram of patient selection and exclusion process

The eligibility of 439 Japanese outpatients on hemodialysis for inclusion in the present study was assessed. Of these, 157 patients did not agree to participate in the study, and 90 patients dropped out between baseline and follow-up surveys. Therefore, 192 hemodialysis patients were finally included in the present study (Fig. 1). The baseline characteristics of the study population are shown in Table 1. The patients consisted of 96 (50%) men, mean age was 64.3 ± 10.3 years, mean time on dialysis was 6.5 ± 7.2 years, and the most common cause of end-stage renal disease was diabetic nephropathy (35.4%) followed by glomerulonephritis (32.8%). Based on the change in physical activity, 47 (24.5%) patients were classed as *becoming less active* group,51 (26.6%) patients were classed as *becoming more active* group, 94 (49.0%) patients were classed as *stable* group. Age and physical activity at baseline and 12 months were different between groups, but no significant differences were observed in other baseline characteristics between groups.

**Table 1:** Demographic and clinical characteristics of hemodialysis patients according to change in physical activity

	All	*Becoming more active* group	*Stable* group	*Becoming less active* group	*P* value
	(*n* = 192)	(*n* = 51)	(*n* = 94)	(*n* = 47)	
Age, years	64.3 ± 10.3	65.6 ± 10.7	63.0 ± 9.6	67.6 ± 10.2	< 0.01
Male,%	96 (50.0%)	23 (45.1%)	55 (58.5%)	18 (38.3%)	0.06
Time on hemodialysis, years	6.5 ± 7.2	5.9 ± 6.7	6.4 ± 6.9	7.4 ± 8.3	0.59
Body mass index, kg/m^2^	21.3 ± 3.3	21.5 ± 3.3	21.4 ± 3.2	20.9 ± 3.5	0.62
Primary kidney disease, %					0.30
Glomerulonephritis	63 (32.8%)	14 (27.5%)	28 (29.8%)	21 (44.7%)	
Diabetes	68 (35.4%)	23 (45.1%)	29 (30.9%)	16 (34.0%)	
Other	24 (12.5%)	7 (13.7%)	12 (12.8%)	4 (8.5%)	
Unknown	20 (10.4%)	3 (5.9%)	14 (14.9%)	3 (6.4%)	
Hypertension	17 (8.8%)	4 (7.8%)	10 (10.6%)	3 (6.4%)	
Comorbidities, %					
Diabetes	84 (43.8%)	25 (49.0%)	36 (38.3%)	23 (48.9%)	0.39
Cerebrovascular accident/TIA	66 (34.4%)	17 (33.3%)	13 (13.8%)	36 (76.6%)	0.02
atherosclerotic heart disease	50 (26.0%)	15 (29.4%)	17 (18.1%)	18 (38.3%)	0.04
Congestive heart failure	24 (12.5%)	10 (19.6%)	10 (10.6%)	4 (8.5%)	0.20
Comorbidity index, score	5.0 ± 3.2	5.7 ± 3.4	4.6 ± 3.0	5.0 ± 3.3	0.13
Serum albumin, g/dL	4.0 ± 0.3	4.0 ± 0.4	4.0 ± 0.3	3.9 ± 0.4	0.62
Physical activity, steps					
Baseline	4421 ± 3048	2951 ± 2300	5261 ± 3274	4335 ± 2703	<0.01
12 months	4291 ± 3211	5028 ± 3817	4929 ± 3003	2214 ± 1701	<0.01

Values are shown as the mean ± SD or *n* (%)

*TIA* transient ischemic attack, *SD* standard deviation

### Survival probability and baseline physical activity

Forty patients died during follow-up over a period of up to 7 years (infection, *n* = 6; cardiovascular disease, *n* = 18; cancer, *n* = 2; cerebral vascular disease, *n* = 2; other causes, *n* = 3; and unknown causes, *n* = 9). The overall follow-up period ranged from 1 to 84 months.

**Fig. 2:**
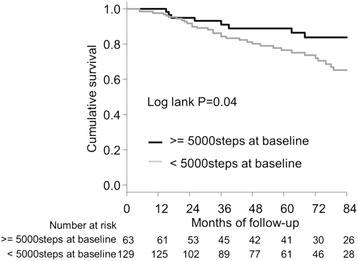
Kaplan-Meier survival analysis of all-cause mortality by baseline physical activity in hemodialysis patients

The 7-year cumulative survival rate were 83.7% in the group of ≥5000 steps of physical activity and 65.2% in the <5000 steps (*P* = 0.04) (Fig. 2). This finding indicates superior survival in patients with greater baseline physical activity.

### Effects of changes in physical activity on survival

**Fig. 3:**
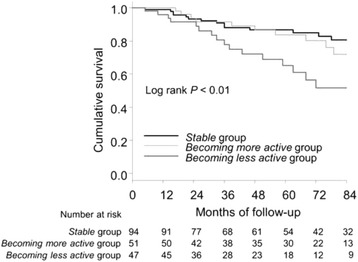
Kaplan-Meier survival analysis of all-cause mortality by changes in physical activity in hemodialysis patients

The median follow-up periods in *becoming less active* group, *stable* group, and *becoming more active* group were 44, 63, and 65 months, respectively (Fig. 3). There were 16 (34.0%) deaths among *becoming less active* group, 14 (14.9%) deaths among *stable* group, and 10 (19.6%) deaths among *becoming more active* group during 7 years. Two in the *becoming less active* group had died, 1 in the *stable* group had died during the first year. On the other hand, all participants in *being more active* group were alive after the first year. The cumulative survival rate in the *becoming less active* group was significantly lower than those in the other groups.

**Fig. 4:**
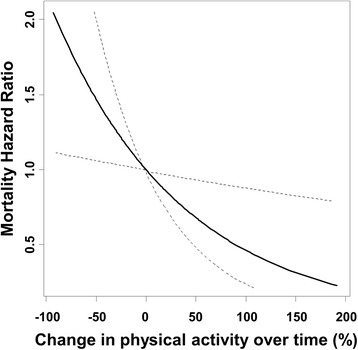
Cubic spline survival analyses exhibiting the association between change in physical activity and mortality. * Adjusted by age, sex, time on hemodialysis, body mass index, primary kidney disease, comorbidity index, serum albumin, and baseline physical activity

Table 2 presents the results of univariate and multivariate Cox proportional hazard analyses for all-cause mortality in the present study population. The crude hazard ratios for all-cause mortality in the *becoming less active* group and *stable* group were 2.21 (95% confidence interval (CI): 1.00–4.89; *P* = 0.05) and 0.70 (95% CI: 0.34–1.74; *P* = 0.54), respectively, compared to the *becoming more active* group. Following adjustment for age, sex, time on hemodialysis, body mass index, primary kidney disease, atherosclerotic heart disease, congestive heart failure, cerebrovascular accident/TIA, diabetes, serum albumin, and baseline physical activity, the hazard ratios for all-cause mortality in the *becoming less active* group and *stable* group were 2.73 (95% CI: 1.12–6.62; *P* = 0.03) and 1.41 (95% CI: 0.53–3.67; *P* = 0.49), respectively, compared to the *becoming more active* group (Model 1). In the Model 2, we adjusted the effect of comorbidity index, which was consisted of cause of end-stage renal disease and 11 comorbidities. Following adjustment for the effects of these covariates, the hazard ratios for all-cause mortality in the *becoming less active* group and *stable* group were 3.68 (95% CI, 1.55–8.78; *P* < 0.01) and 1.93 (95% CI: 0.75–4.99; *P* = 0.17), respectively, compared to the *becoming more active* group. These results indicated a significant association between the yearly change in physical activity and poor prognosis of hemodialysis patients independent of not only patient characteristics but also baseline physical activity. Graphical visualization of the Cox proportional hazard model using the restricted cubic spline procedure indicated a significant relation between change in physical activity and all-cause mortality risk in the present study population (Fig. 4).

**Table 2:** Cox proportional hazards model for all-cause mortality

Parameter	Univariate analysis^a^			Multivariate analysis: Model 1^b^			Multivariate analysis: Model 2^c^		
	Hazard ratio (95% CI)		*P* value	Hazard ratio (95% CI)		*P* value	Hazard ratio (95% CI)		*P* value
Change in physical activity									
*Becoming more active* group	1.00			1.00			1.00		
*Stable* group	0.70	(0.34–1.74)	0.54	1.41	(0.53–3.67)	0.49	1.93	(0.75–4.99)	0.17
*Becoming less active* group	2.21	(1.00–4.89)	0.05	2.73	(1.12–6.62)	0.03	3.68	(1.55–8.78)	< 0.01

Analyses were performed using Cox proportional hazards regression

*CI* confidence interval

^a^Unadjusted by characteristic factors on survival

^b^Adjusted by age, sex, time on hemodialysis, body mass index, primary kidney disease, atherosclerotic heart disease, congestive heart failure, cerebrovascular accident/transient ischemic attack, diabetes, serum albumin, and baseline physical activity

^c^Adjusted by age, sex, time on hemodialysis, body mass index, primary kidney disease, comorbidity index, serum albumin, and baseline physical activity

## Discussion

The present study was performed to examine all-cause mortality rate in a cohort of 192 hemodialysis patients. Forty (20.8%) patients died during the observation period lasting up to 7 years, with cardiovascular disease as the leading cause of death. Almost one quarter of the patients showed a decline in physical activity over time, which was significantly related to elevated mortality risk independent of patient characteristics and baseline physical activity. To our knowledge, this is the first report of an association between change in physical activity and mortality in hemodialysis patients. Our findings suggest that it is important to prevent a decline in physical activity over time in hemodialysis patients to improve their prognosis.

Only a few studies have examined changes in physical activity evaluated with an accelerometer or pedometer and mortality. Yates et al. reported that a decrease and an increase of approximately 25% in steps per day from baseline were noted in 25.2 and 24.9% of people with impaired glucose tolerance, respectively, at 1 year follow-up [7]. In the present study, 24.5% and 26.6% of hemodialysis patients experienced a decrease and an increase of 30%, respectively, in physical activity level from baseline. Our findings are consistent with those of the previous study targeting people with chronic illness. In addition, 90 patients dropped out during baseline and follow-up surveys in the present study. If an assessment of yearly changes in physical activity levels of these patients was possible, the rate of patients who experienced a > 30% decrease in physical activity might have been higher.

There are several possible explanations for the association between decline in physical activity over time and poor prognosis in hemodialysis patients observed in the present study. First, arteriosclerotic disease is the main cause of death in patients on hemodialysis, as also indicated in this study. Previous reports indicated that engaging in physical activity or low-intensity walking exercise improved risk factors for arteriosclerotic disease in hemodialysis patients, i.e., hypertension, arterial stiffness, plasma triglyceride and cholesterol levels, cardiac autonomic system dysfunction, and reduced maximal oxygen consumption [20–28]. Therefore, reductions in level of physical activity over time in such patients could lead to elevated risk of arteriosclerosis. The results of the present study suggested that changes in physical activity may be related to risk factors for cardiovascular disease. However, the associations could have been confounded by lifestyle-related factors that were either not measured or were measured inaccurately. In addition, the observational design of this study did not allow us to establish a causal link between change in physical activity and all-cause mortality rate. Second, patients whose physical activity at 12 months could not be determined due to severe condition were excluded from the analyses in the present study. However, we may have failed to exclude some patients with slight deterioration of overall condition that did not reach a level requiring medical treatment. Regardless of these possibilities, this study indicated that it is important to evaluate changes in physical activity among hemodialysis patients to predict their prognosis and to allow better disease management.

Tudor-Locke et al. reported that 5000 steps per day at baseline are a “borderline for mortality” in older adults [18]. In this study, participants indicates superior survival in patients with > = 5000 steps per day. Previous studies indicated an association between baseline physical activity and survival in hemodialysis patients [10, 12, 13]. Therefore, the results of the present observational study extend previous research by suggesting that baseline physical activity and changes in physical activity are both important and independent determinants of all-cause mortality in hemodialysis patients.

Byberg et al. examined the effects of changes in physical activity on prognosis in a large community dwelling population-based cohort [29], and reported a higher mortality risk in people with a decline in physical activity over time from a high to low or moderate level, even if their baseline levels of physical activity was high. In addition, Yates et al. reported that each increase of 2000 steps per day from baseline to 12 months was significantly associated with an additional 8% difference in the cardiovascular event rate in people with impaired glucose tolerance [7]. These previous reports indicating relations between decline in physical activity over time and poor prognosis in myocardial infarction patients, diabetes, and community dwelling populations were similar to the results of the present study [17, 29–31]. As a more representative measure of physical activity, especially in hemodialysis patients, not only baseline physical activity but also change in physical activity should be evaluated. In addition, we measured physical activity with an accelerometer rather than by interview or questionnaire; determination of physical activity by interview or questionnaire has been used frequently due to its ease, but use of an accelerometer during routine daily activities is recommended in hemodialysis patients [32], as previous studies determined physical activity of hemodialysis patients using accelerometers or pedometers [1, 3]. The present study indicated the association between changes in physical activity measured objectively using an accelerometer and mortality in hemodialysis patients.

Despite evidence regarding the health benefits of physical activity and exercise, most hemodialysis patients are not sufficiently physically active. There are several means of increasing physical activity, including the use of a pedometer that reports the change in number of steps walked per day. Adults were reported to show an increase in physical activity by 26.9% over baseline over 18 weeks of pedometer use [33]. Early studies also indicated that use of a pedometer motivated hemodialysis patients to increase their level of physical activity [34, 35]. The level of physical activity can also be increased by exercise training. Frailty is a primary pathway to disability, defined as a pathological condition that results in a constellation of signs and symptoms and is characterized by high susceptibility to adverse health outcomes, impending decline in physical function, and high risk of death [36], which is common in hemodialysis patients [37–41]. Frailty in such patients is a major factor preventing the adoption of an active lifestyle. However, there is a great deal of evidence that exercise training is beneficial for older adults at high risk of frailty [42]. Hence, it is possible that the levels of physical activity can be increased by ameliorating frailty. In fact, Chen et al. reported increases in physical activity among hemodialysis patients that participated in a low-intensity intradialytic exercise program accompanied by improvement in physical performance [43]. Therefore, most hemodialysis patients should change from a sedentary to a non-sedentary lifestyle to improve their long-term prognosis.

This study had a number of limitations. First, in this study, the patients in *becoming less active* group were more likely older, and had a greater prevalence of cerebrovascular accident/TIA and atherosclerotic heart disease, and had higher physical activity level at baseline than the others. Although we should perform subgroup analyses according to age, the prevalence of these comorbidities and baseline physical activity level in order to adjust the effects of differences in baseline characteristics between 3 groups, we abandon the analyses because of the small sample size. Instead of this, we analyzed the association between change in physical activity and mortality risks using Cox proportional hazards regression models adjusting for the differences in baseline characteristics of the study participants. And, this is one of only a few studies reported to date involving examination of objectively measured physical activity using an accelerometer or pedometer in hemodialysis patients. Further large-scale observational studies are needed. Second, we excluded patients that required assistance with walking and did not complete the evaluation of physical activity at 12 months because of adverse events or lower adherence. Therefore, the comorbidities in the participants were mild, which should be taken into consideration when generalizing our results to patients with more severe limitations. Third, we measured participant physical activity over the course of 4-day monitoring. Although previous studies recommended the use of data from at least 3- to 4-day monitoring to reliably predict total physical activity behavior over long term periods [44, 45], our survey period was short. In addition, the lack of data that could interfere with the measurement of true physical activity (e.g., season, social factors) might have introduced bias in our assessment. Forth, one of the five participants was classified as “becoming less active” (24.5%). We could not provide an explanation as to why physical activity levels decreased in many patients after 12 months, as we did not collect information relating to factors that could limit physical activities in hemodialysis patients, such as higher age, comorbidities, physical function, body pain, exacerbation of dialysis-related symptoms, depression symptoms, adherence to exercise, and cohabiting family. Accordingly, an observational study will be necessary to further assess the causes of sedentary lifestyle among hemodialysis patients, including factors associated with changes in physical activity over time. Finally, although we showed that mortality risk was higher in patients with a decline in physical activity over time compared with the other groups, the mechanisms underlying these observations remain to be elucidated.

## Conclusions

Reductions in physical activity were significantly associated with poor prognosis independent of not only patient characteristics but also baseline physical activity. Therefore, improved prognosis in hemodialysis patients requires means of preventing a decline in physical activity over time.

## Abbreviations

ANOVA: Analysis of variance
CI: Confidence interval
SD: Standard deviation
TIA: Transient ischemic attack

## References

[1] Johansen KL, Chertow GM, Ng AV, Mulligan K, Carey S, Schoenfeld PY, Kent-Braun JA (2000). Physical activity levels in patients on hemodialysis and healthy sedentary controls. Kidney Int.

[2] Johansen KL, Chertow GM, Kutner NG, Dalrymple LS, Grimes BA, Kaysen GA (2010). Low level of self-reported physical activity in ambulatory patients new to dialysis. Kidney Int.

[3] Zamojska S, Szklarek M, Niewodniczy M, Nowicki M (2006). Correlates of habitual physical activity in chronic haemodialysis patients. Nephrol Dial Transplant.

[4] Avesani CM, Trolonge S, Deleaval P, Baria F, Mafra D, Faxen-Irving G, Chauveau P, Teta D, Kamimura MA, Cuppari L (2012). Physical activity and energy expenditure in haemodialysis patients: an international survey. Nephrol Dial Transplant.

[5] Stack AG, Molony DA, Rives T, Tyson J, Murthy BV (2005). Association of physical activity with mortality in the US dialysis population. Am J Kidney Dis.

[6] Kyu HH, Bachman VF, Alexander LT, Mumford JE, Afshin A, Estep K, Veerman JL, Delwiche K, Iannarone ML, Moyer ML (2016). Physical activity and risk of breast cancer, colon cancer, diabetes, ischemic heart disease, and ischemic stroke events: systematic review and dose-response meta-analysis for the global burden of disease study 2013. BMJ.

[7] Yates T, Haffner SM, Schulte PJ, Thomas L, Huffman KM, Bales CW, Califf RM, Holman RR, McMurray JJV, Bethel MA (2014). Association between change in daily ambulatory activity and cardiovascular events in people with impaired glucose tolerance (NAVIGATOR trial): a cohort analysis. Lancet.

[8] Miura Y, Fukumoto Y, Miura T, Shimada K, Asakura M, Kadokami T, Ando S, Miyata S, Sakata Y, Daida H (2013). Impact of physical activity on cardiovascular events in patients with chronic heart failure. A multicenter prospective cohort study. Circ J.

[9] O'Hare AM, Tawney K, Bacchetti P, Johansen KL (2003). Decreased survival among sedentary patients undergoing dialysis: results from the dialysis morbidity and mortality study wave 2. Am J Kidney Dis.

[10] Tentori F, Elder SJ, Thumma J, Pisoni RL, Bommer J, Fissell RB, Fukuhara S, Jadoul M, Keen ML, Saran R (2010). Physical exercise among participants in the dialysis outcomes and practice patterns study (DOPPS): correlates and associated outcomes. Nephrol Dial Transplant.

[11] Lopes AA, Lantz B, Morgenstern H, Wang M, Bieber BA, Gillespie BW, Li Y, Painter P, Jacobson SH, Rayner HC (2014). Associations of self-reported physical activity types and levels with quality of life, depression symptoms, and mortality in hemodialysis patients: the DOPPS. Clin J Am Soc Nephrol.

[12] Johansen KL, Kaysen GA, Dalrymple LS, Grimes BA, Glidden DV, Anand S, Chertow GM (2013). Association of physical activity with survival among ambulatory patients on dialysis: the comprehensive dialysis study. Clin J Am Soc Nephrol.

[13] Matsuzawa R, Matsunaga A, Wang G, Kutsuna T, Ishii A, Abe Y, Takagi Y, Yoshida A, Takahira N (2012). Habitual physical activity measured by accelerometer and survival in maintenance hemodialysis patients. Clin J Am Soc Nephrol.

[14] Liu J, Huang Z, Gilbertson DT, Foley RN, Collins AJ (2010). An improved comorbidity index for outcome analyses among dialysis patients. Kidney Int.

[15] Schneider PL, Crouter SE, Lukajic O, Bassett DR (2003). Accuracy and reliability of 10 pedometers for measuring steps over a 400-m walk. Med Sci Sports Exerc.

[16] Crouter SE, Schneider PL, Karabulut M, Bassett DR (2003). Validity of 10 electronic pedometers for measuring steps, distance, and energy cost. Med Sci Sports Exerc.

[17] Dwyer T, Ponsonby AL, Ukoumunne OC, Pezic A, Venn A, Dunstan D, Barr E, Blair S, Cochrane J, Zimmet P (2011). Association of change in daily step count over five years with insulin sensitivity and adiposity: population based cohort study. BMJ.

[18] Tudor-Locke C, Craig CL, Aoyagi Y, Bell RC, Croteau KA, De Bourdeaudhuij I, Ewald B, Gardner AW, Hatano Y, Lutes LD (2011). How many steps/day are enough? For older adults and special populations. Int J Behav Nutr Phys Act.

[19] Kanda Y (2013). Investigation of the freely available easy-to-use software ‘EZR’ for medical statistics. Bone Marrow Transplant.

[20] Tozawa M, Iseki K, Iseki C, Takishita S (2002). Pulse pressure and risk of total mortality and cardiovascular events in patients on chronic hemodialysis. Kidney Int.

[21] Shoji T, Tsubakihara Y, Fujii M, Imai E (2004). Hemodialysis-associated hypotension as an independent risk factor for two-year mortality in hemodialysis patients. Kidney Int.

[22] Hase H, Tsunoda T, Tanaka Y, Takahashi Y, Imamura Y, Ishikawa H, Inishi Y, Joki N (2006). Risk factors for de novo acute cardiac events in patients initiating hemodialysis with no previous cardiac symptom. Kidney Int.

[23] Shoji T, Masakane I, Watanabe Y, Iseki K, Tsubakihara Y (2011). Elevated non-high-density lipoprotein cholesterol (non-HDL-C) predicts atherosclerotic cardiovascular events in hemodialysis patients. Clin J Am Soc Nephrol.

[24] Sarnak MJ, Levey AS, Schoolwerth AC, Coresh J, Culleton B, Hamm LL, McCullough PA, Kasiske BL, Kelepouris E, Klag MJ (2003). Kidney disease as a risk factor for development of cardiovascular disease: a statement from the American Heart Association councils on kidney in cardiovascular disease, high blood pressure research, clinical cardiology, and epidemiology and prevention. Circulation.

[25] Matsuzawa R, Matsunaga A, Kutsuna T, Ishii A, Abe Y, Yoneki K, Harada M, Ishibashi M, Takeuchi Y, Yoshida A (2014). Association of habitual physical activity measured by an accelerometer with high-density lipoprotein cholesterol levels in maintenance hemodialysis patients. ScientificWorldJournal.

[26] Goldberg AP, Geltman EM, Gavin JR, Carney RM, Hagberg JM, Delmez JA, Naumovich A, Oldfield MH, Harter HR (1986). Exercise training reduces coronary risk and effectively rehabilitates hemodialysis patients. Nephron.

[27] Hagberg JM, Goldberg AP, Ehsani AA, Heath GW, Delmez JA, Harter HR (1983). Exercise training improves hypertension in hemodialysis patients. Am J Nephrol.

[28] Rosa CS, Bueno DR, Souza GD, Gobbo LA, Freitas IF, Sakkas GK, Monteiro HL (2015). Factors associated with leisure-time physical activity among patients undergoing hemodialysis. BMC Nephrol.

[29] Byberg L, Melhus H, Gedeborg R, Sundstrom J, Ahlbom A, Zethelius B, Berglund LG, Wolk A, Michaelsson K (2009). Total mortality after changes in leisure time physical activity in 50 year old men: 35 year follow-up of population based cohort. BMJ.

[30] Petersen CB, Gronbaek M, Helge JW, Thygesen LC, Schnohr P, Tolstrup JS (2012). Changes in physical activity in leisure time and the risk of myocardial infarction, ischemic heart disease, and all-cause mortality. Eur J Epidemiol.

[31] Wolin KY, Patel AV, Campbell PT, Jacobs EJ, McCullough ML, Colditz GA, Gapstur SM (2010). Change in physical activity and colon cancer incidence and mortality. Cancer Epidemiol Biomarkers Prev.

[32] Johansen KL, Painter P, Kent-Braun JA, Ng AV, Carey S, Da Silva M, Chertow GM (2001). Validation of questionnaires to estimate physical activity and functioning in end-stage renal disease. Kidney Int.

[33] Bravata DM, Smith-Spangler C, Sundaram V, Gienger AL, Lin N, Lewis R, Stave CD, Olkin I, Sirard JR (2007). Using pedometers to increase physical activity and improve health. JAMA.

[34] Nowicki M, Murlikiewicz K, Jagodzinska M (2010). Pedometers as a means to increase spontaneous physical activity in chronic hemodialysis patients. J Nephrol.

[35] Bohm C, Stewart K, Onyskie-Marcus J, Esliger D, Kriellaars D, Rigatto C (2014). Effects of intradialytic cycling compared with pedometry on physical function in chronic outpatient hemodialysis: a prospective randomized trial. Nephrol Dial Transplant.

[36] Fried LP, Tangen CM, Walston J, Newman AB, Hirsch C, Gottdiener J, Seeman T, Tracy R, Kop WJ, Burke G (2001). Frailty in older adults, evidence for a phenotype. J Gerontol A Biol Sci Med Sci.

[37] Bao Y, Dalrymple L, Chertow GM, Kaysen GA, Johansen KL (2012). Frailty, dialysis initiation, and mortality in end-stage renal disease. Arch Intern Med.

[38] Johansen KL, Chertow GM, Jin C, Kutner NG (2007). Significance of frailty among dialysis patients. J Am Soc Nephrol.

[39] McAdams-DeMarco MA, Law A, Salter ML, Boyarsky B, Gimenez L, Jaar BG, Walston JD, Segev DL (2013). Frailty as a novel predictor of mortality and hospitalization in individuals of all ages undergoing hemodialysis. J Am Geriatr Soc.

[40] McAdams-DeMarco MA, Suresh S, Law A, Salter ML, Gimenez LF, Jaar BG, Walston JD, Segev DL (2013). Frailty and falls among adult patients undergoing chronic hemodialysis: a prospective cohort study. BMC Nephrol.

[41] Matsuzawa R, Matsunaga A, Wang G, Yamamoto S, Kutsuna T, Ishii A, Abe Y, Yoneki K, Yoshida A, Takahira N (2014). Relationship between lower extremity muscle strength and all-cause mortality in Japanese patients undergoing dialysis. Phys Ther.

[42] Liu CK, Fielding RA (2011). Exercise as an intervention for frailty. Clin Geriatr Med.

[43] Chen JL, Godfrey S, Ng TT, Moorthi R, Liangos O, Ruthazer R, Jaber BL, Levey AS, Castaneda-Sceppa C (2010). Effect of intra-dialytic, low-intensity strength training on functional capacity in adult haemodialysis patients: a randomized pilot trial. Nephrol Dial Transplant.

[44] Matthews CE, Ainsworth BE, Thompson RW, Bassett DR (2002). Sources of variance in daily physical activity levels as measured by an accelerometer. Med Sci Sports Exerc.

[45] Trost SG, McIver KL, Pate RR (2005). Conducting accelerometer-based activity assessments in field-based research. Med Sci Sports Exerc.

